# Percutaneous periarticular multi-drug injection at one day after total knee arthroplasty as a component of multimodal pain management: a randomized control trial

**DOI:** 10.1186/s12891-019-2451-1

**Published:** 2019-02-08

**Authors:** Takuya Iseki, Sachiyuki Tsukada, Motohiro Wakui, Kenji Kurosaka, Shinichi Yoshiya

**Affiliations:** 1 Department of Orthopaedic Surgery, Nekoyama Miyao Hospital, 14-7 Konan, Chuo-ku, Niigata, Niigata 950-1151 Japan; 2 Department of Orthopaedic Surgery, Hyogo College of Medicine, 1-1 Mukogawa-cho, Nishinomiya City, Hyogo 663-8501 Japan; 3 Department of Orthopaedic Surgery, Hokusuikai Kinen Hospital, 3-2-1 Higashihara, Mito, Ibaraki, 310-0035 Japan

**Keywords:** Knee, Primary arthroplasty, Multimodal, Pain management, Opioid

## Abstract

**Background:**

Although intraoperative periarticular multi-drug injection has been used for postoperative pain control after total knee arthroplasty (TKA), the injection has the inherent shortcoming of limited acting time. This randomized controlled trial was performed to assess whether adding percutaneous periarticular multi-drug injection at the day following TKA would improve the postoperative pain relief.

**Methods:**

A total of 43 participants were randomly assigned to receive additional periarticular injection at 08:30, postoperative day 1 or no additional injection. The multi-drug solution including 40 mg of methylprednisolone, 150 mg of ropivacaine, and 0.1 mg of epinephrine was infiltrated into the muscle belly of the vastus medialis. In both groups, patients were treated with intraoperative periarticular multi-drug injection and postoperative intravenous and oral nonsteroidal anti-inflammatory drugs. We did not use any narcotic pain medications postoperatively. The primary outcome was the patients’ global assessment of postoperative pain at rest measured using a visual analog scale (VAS) and quantified as the area under the curve (AUC) of serial assessments until 20:00, postoperative day 5.

**Results:**

The mean AUC for the postoperative pain VAS at rest was 1616 ± 1191 in patients received the additional periarticular injection versus 2808 ± 1494 in those received no injection (mean difference, − 1192; 95% confidence interval, − 2043 to − 340; *p* = 0.007). No wound complication or surgical site infection was observed in either groups.

**Conclusions:**

Adding percutaneous periarticular multi-drug injection at the day following TKA may provide better postoperative pain relief. Further studies are needed to confirm the safety of the percutaneous injection.

**Trial registration:**

University Hospital Medical Information Network UMIN000029003. Registered 5 September 2017.

## Background

Pain is severe after total knee arthroplasty (TKA) [1]. Multimodal pain management including periarticular multi-drug injection has provided an improvement on the pain in the early postoperative period of TKA [1, 2]. During the early postoperative period, the pain score below the threshold value of patient acceptable symptom state (PASS), which is defined as a symptom state that a patient considers acceptable [3], was reported in patients treated with intraoperative periarticular injections including opioid, local anesthetic, corticosteroid, non-steroidal anti-inflammatory drugs, and epinephrine [4, 5]. However, the pain score elevated 24 h after TKA [4, 5]. The rebounding pain after the early postoperative period remains a critical issue in patients treated with multimodal pain management [6].

Several investigators tried to add continuous intraarticular infusion to multimodal pain management including periarticular multi-drug injection [2, 7–10]. However, Ali et al. reported that the surgical site infection rate increased with use of the continuous intraarticular infusion technique [10]. A percutaneous periarticular multi-drug injection technique at the day following TKA was developed, in which the infusion catheter was not used.

We conducted a prospective, randomized, open-label trial to investigate the impact of the percutaneous periarticular multi-drug injection at the day following TKA on multimodal pain management for TKA. The hypothesis of this study was that the postoperative pain score would be lower in patients that received the percutaneous periarticular multi-drug injection.

## Methods

### Study design

We did a single-center, two-arm, parallel group, randomized, open-label trial with 1:1 treatment allocation. The randomized controlled trial was approved by the institutional review board on 3 September 2017. Written informed consent was obtained from eligible participants. Before the onset of participant enrolment, the trial was registered as a randomized controlled trial titled “Percutaneous periarticular analgesic injection at one day after total knee arthroplasty as a component of multimodal pain management: a randomized control trial” with the University Hospital Medical Information Network (UMIN) that is accepted registration by the International Committee of Medical Journal Editors (ICMJE).

### Study population

Patients were recruited between October 2017 and March 2018 from a single orthopedic clinic (Nekoyama Miyao Hospital, Niigata, Japan). We prespecified the inclusion criteria of this randomized controlled trial as patients older than 20 years of age and scheduled for TKA of the unilateral knee. We prespecified the exclusion criteria as below: (1) patients scheduled for revision TKA, (2) scheduled for TKA combined with implant removal, (3) having allergy or intolerance to one of the study drugs, and (4) having poorly controlled diabetic mellitus defined as hemoglobin A1c with levels over 7.0%. Patients were not excluded based on their primary disease to maintain generalizability.

### Randomization

Patients were randomly assigned to receive either percutaneous periarticular multi-drug injection at the day following TKA or no injection. The randomization schedule was generated by an independent investigator (KK) who did not take part in surgery or the assessment of outcome, by means of a computer-derived random-number sequence. The investigator created the randomization sequence by permuted block randomization with a block size of 4 and a 1:1 allocation generated using a computer software (R, The R Foundation for Statistical Computing, Vienna, Austria). When a patient participated in the trial, the generated randomized number was assigned accordingly. Patients with even numbers were allocated to the group scheduled to receive percutaneous periarticular multi-drug injection at the day following TKA, and those with odd numbers were allocated to receive no injection.

### Pre- and postoperative medication

The pre- and postoperative medications were identical in both groups except for the percutaneous periarticular multi-drug injection at the day following TKA. We routinely performed intraoperative periarticular injection for the perioperative pain management. The multi-drug solution of the injection included methylprednisolone 40 mg (Sol Mercort; Fuji, Toyama, Japan) [1 mL] as corticosteroid, 7.5 mg/mL ropivacaine (Anapeine; AstraZeneca, Osaka, Japan) [40 mL] as local anesthetics, 10 mg/mL morphine hydrochloride hydrate (Takeda, Osaka, Japan) [0.8 mL] as opioid, 1.0 mg/mL epinephrine (Bosmin; Daiichi-Sankyo, Tokyo, Japan) [0.3 mL], and 50 mg of ketoprofen (Capisten; Kissei, Matsumoto, Japan) [2.5 mL] as non-steroidal anti-inflammatory drugs [4, 5]. The solution was mixed with 15.4 mL of saline to a combined volume of 60 mL. Intravenous 50 mg of flurbiprofen axetil (Ropion; Kaken, Tokyo, Japan) was given 1 h after turning back to the hospital ward. From the day following TKA, oral 60 mg of loxoprofen (Surinofen; Aska, Tokyo, Japan) was given three times per day. Study protocol permitted 25 mg of diclofenac sodium suppository (Adefuroniczupo; Teva, Nagoya, Japan) as rescue therapy. Opioid usage was prohibited apart from the morphine hydrochloride hydrate contained in the intraoperative periarticular injection.

### Surgical procedure and rehabilitation program

All TKAs were carried out in a unified manner. Surgeries were managed under lumbar analgesia with 2.0 to 2.8 mL of 0.5% bupivacaine (Marcaine; AstraZeneca). One of three surgeons (TI, ST and MW) conducted all surgical procedures. We did not use any pneumatic tourniquets. No drain was applied to any of the patients. We designed an anteromedial straight skin incision that began 4 cm proximal to the patella and ended 1 cm distal to the tibial tuberosity with the knee flexed. We lengthened the skin incision as necessary to adequately expose the knee joint during surgery. A subvastus approach was used for surgical approach. All patients received Scorpio NRG-PS (Stryker Orthopaedics, Mahwah, NJ), a cemented posterior stabilized prosthesis. The postoperative rehabilitation regimens were the same for both groups and started from the afternoon on the day following surgery.

### Interventions

The study treatments were percutaneous periarticular multi-drug injection at the day following TKA (additional periarticular injection group) and no injection (no additional injection group). The percutaneous periarticular multi-drug injection was routinely performed at 08:30 AM regardless of the time that TKA was performed.

**Fig. 1:**
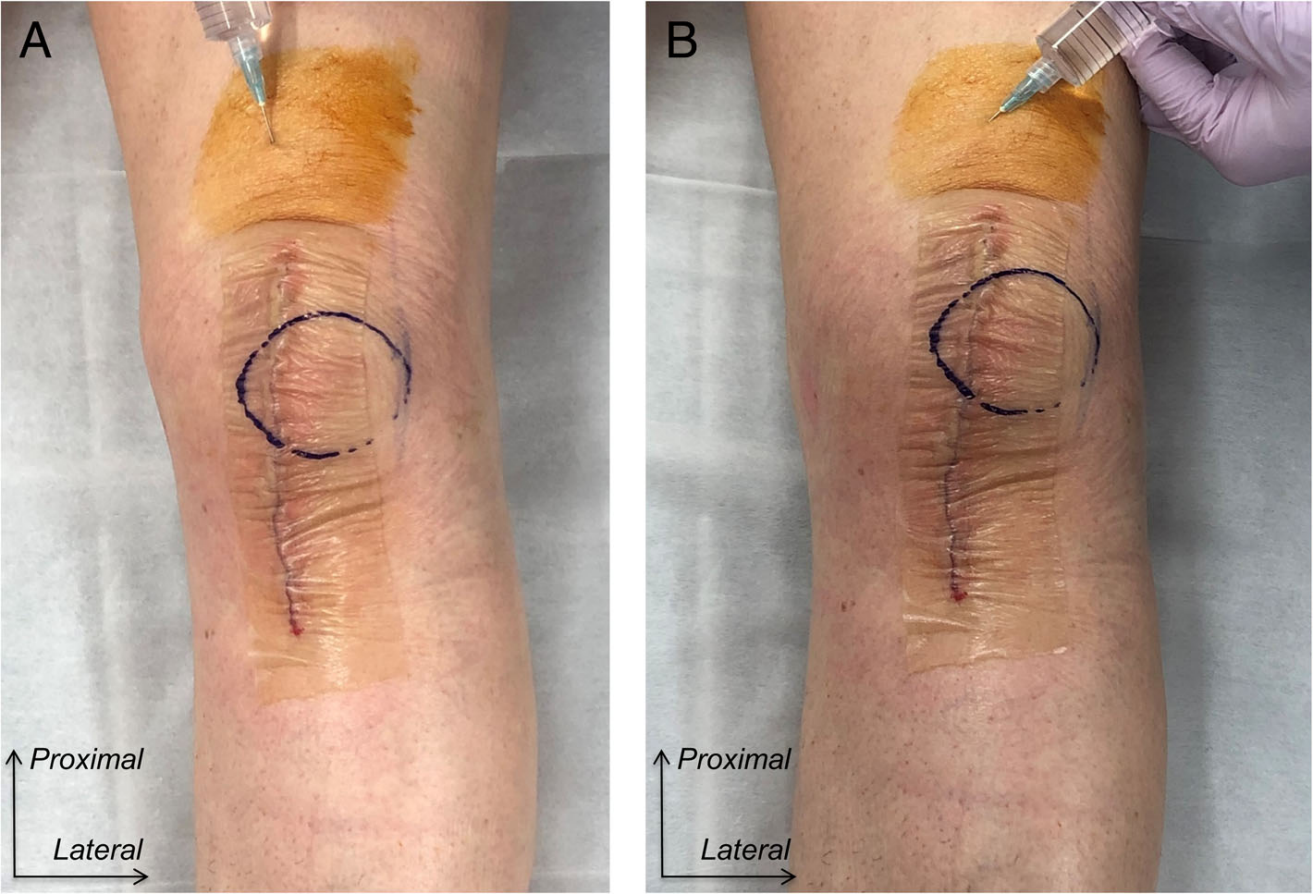
The infiltration technique for the percutaneous periarticular multi-drug injection into the muscle belly of the vastus medialis performed at the day following total knee arthroplasty for the left knee. **a**. Infiltration into the muscle belly of the vastus medialis just medial to the quadriceps tendon. **b**. Infiltration into the muscle belly of vastus medialis at a more medial site of the first infiltration. Note that we injected the solution into neither joint space of the knee nor subcutaneous tissue

In the additional periarticular injection group, patients received percutaneous periarticular multi-drug injection including methylprednisolone 40 mg [1 mL], ropivacaine 150 mg [20 mL], and epinephrine 0.1 mg [0.1 mL] at the day following TKA. The injection was performed at approximately 3 cm above the superior border of the antero-medial skin incision using 23-gauge needle with the knee extended (Fig. 1). After disinfecting skin using povidone iodine and ethanol, a total of 21.1 mL of multi-drug solution was injected into the muscle belly of vastus mediallis. We were careful not to inject the solution into joint space of the knee or subcutaneous tissue. The injection point was a single location. After insertion of the needle, we moved the needle tip to the aiming site without pulling out the needle from the skin. First, we infiltrated 11.1 mL of solution into the muscle belly of the vastus medialis just medial to the quadriceps tendon (Fig. 1a). Second, we moved the needle tip to medial, and infiltrated remaining 10 mL of solution into the muscle belly of vastus medialis at a more medial site than the first infiltration (Fig. 1b).

### Outcome measurements

#### Primary outcome

We prespecified the pain scale at rest measured using VAS score as the primary outcome of this study. The VAS score ranged from 0 mm (indicating no pain) to 100 mm (indicating extreme pain). The VAS score was measured 1 day after surgery at 12:00 and 20:00, and 2, 3, 4, and 5 days after surgery at 6:00, 12:00, and 20:00. The measurement of VAS score at rest was performed during the hospital stay.

#### Secondary outcome

The prespecified secondary outcomes of this study were postoperative level of pain during activity, range of motion of the knee, and complications. We defined the strongest pain experienced during physical therapy exercise on a day as the VAS score during activity. We also recorded the amount of the consumption of rescue analgesia during the study period. The data was collected from postoperative days 1 to 5.

### Sample size calculation and statistical analyses

A total of 16 patients per group was needed to fulfill the minimal clinically important difference (MCID) of 20 mm in the point-by-point assessment of primary outcome [4, 5], with a type I error rate of 5%, and a type II error rate of 10% (90% power). A standard deviation of 17 mm was determined to the results of our previous randomized controlled trial [6].

Study protocol prescribed that missing data of the primary outcome were filled up by linear interpolation between two valid data. When the missing data did not lie between two valid data, the missing data were filled up by the medians of the treatment groups at the same measurement time.

To compare the cumulative pain VAS score at rest after TKA, we quantified area under the curve (AUC) of each study patient. The calculation was performed as follows: we made a line graph for each patient in which VAS score was on the vertical axis and time after surgery was on the horizontal axis and we divided the area under the graph into a series of trapezoids. The sum of the areas of each of the individual trapezoids was calculated as the AUC [5, 11–13]. The AUC integrates all VAS scores at rest measured over time [12]. The mean differences in the AUC of the VAS scores at rest until 20:00, postoperative day 5 and 95% confidence intervals were compared between additional periarticular injection group and no additional injection group using Student’s t test.

The serial measurements of pain VAS scores were also analyzed applying repeated-measures analysis of variance to data obtained at each time point to establish whether there were significant differences over time between two groups [14]. We applied Mauchly sphericity test, and corrections were made using the Greenhouse-Geisser test when sphericity was rejected. *P* value for the effect of group, which means the effect of the additional periarticular injection, was assessed.

In addition to statistical significance, the effect size of patient-reported outcomes should be assessed whether they are meaningful in the clinical setting [3]. The VAS score at rest in each time point was also assessed to determine whether they would reach the PASS and MCID. The threshold of PASS was defined as 33 mm according to the study conducted by Myles et al. [3]. The threshold of MCID was determined as 20 mm as mentioned above [4, 5].

Patient demographics and baseline clinical characteristics were compared with Student’s *t* test for continuous variables and the Fisher’s exact test for categorical variables between the study groups. We assessed changes in the VAS scores during activity, the consumption of diclofenac sodium suppository as rescue analgesia, and the range of knee motion between groups using analysis of variance for a repeated-measures analysis of variance. All tests were two-sided. We considered *P* < 0.05 as statistically significant. All statistical analyses were done using R software.

## Results

### Participants

**Fig. 2:**
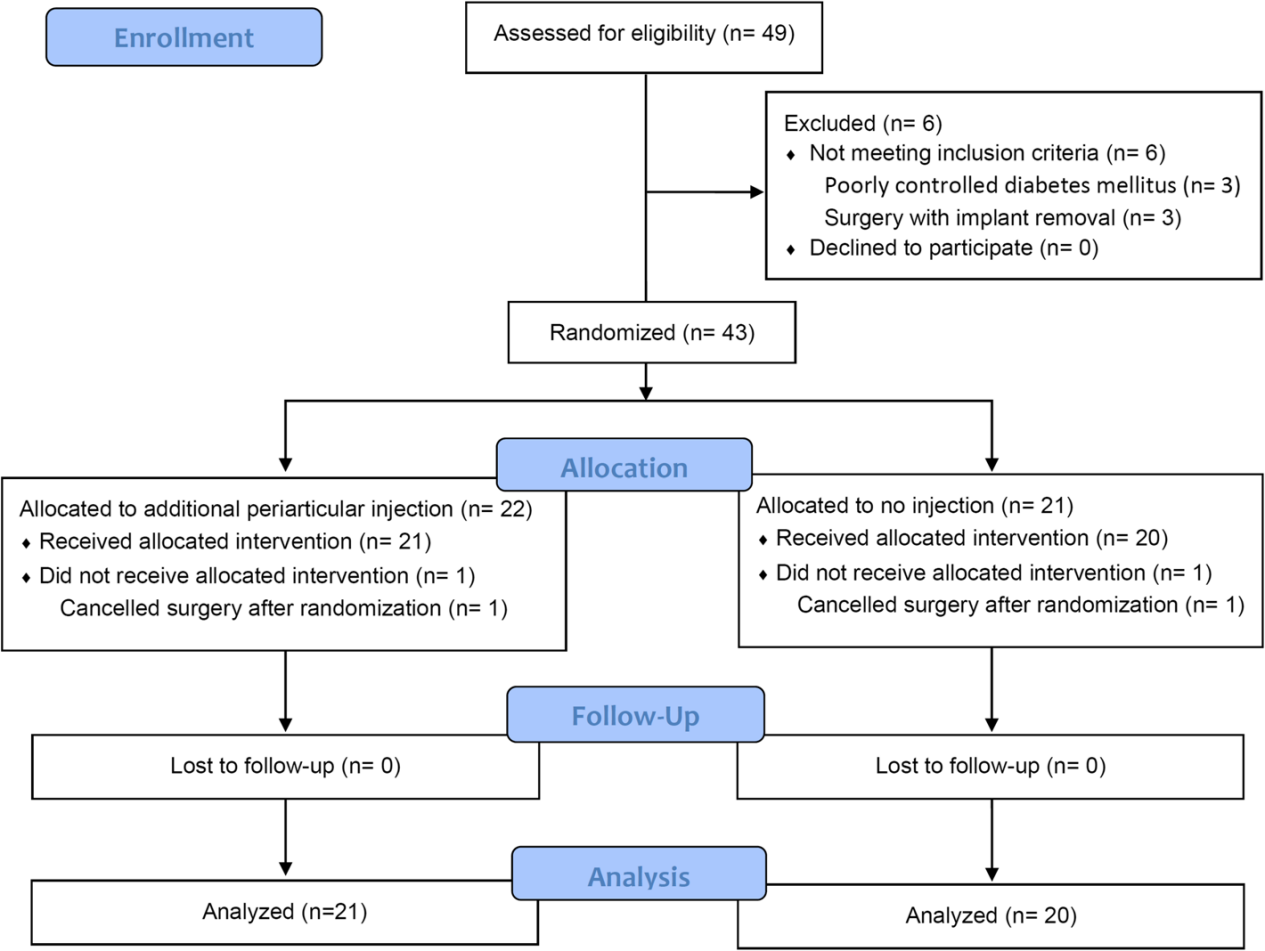
Diagram showing the flow of patients through each stage of the trial

A total of 49 patients underwent unilateral TKA during the study period and were eligible for inclusion in the study. The flowchart presented in Fig. 2 outlines the trial. Of 49 patients screened for eligibility, 43 were randomly assigned to receive an additional periarticular injection or no injection (*n* = 22 and *n* = 21, respectively). Table 1 shows the demographic characteristics of the patients in the two groups. After randomization, we excluded one patient in each group because of cancelled surgery.

**Table 1:** Patient demographics and baseline clinical characteristics

Variable	Additional periarticular injection (22 patients)	No additional injection (21 patients)	*P-*value
Age, years	72 ± 7	76 ± 8	0.12*
Sex (Female/Male)	19/3	15/6	0.28†
Side (Right/Left)	10/12	12/9	0.55†
Height, cm	153 ± 10	152 ± 7	0.69*
Weight, kg	61.3 ± 9.0	60.6 ± 10.6	0.82*
Body mass index, kg/m^2^	26.4 ± 4.1	26.3 ± 3.7	0.92*
Diagnosis (Osteoarthritis/Avascular necrosis)	19/3	16/5	0.46†
History of diabetes mellitus (yes/no)	4/18	3/18	> 0.99†
Preoperative visual analog scale at rest, mm	35 ± 32	40 ± 27	0.61*
Preoperative visual analog scale during activity, mm	39 ± 22	37 ± 22	0.85*
Preoperative flexion angle, degree	127 ± 17	120 ± 18	0.26*
Preoperative extension angle, degree	− 9 ± 9	−9 ± 8	0.99*
Duration of surgery, min	84 ± 10	86 ± 15	0.53*

Results are expressed as means ± standard deviation, unless stated otherwise

*P-values were determined with Student’s *t* test

† *P*-values were determined with Fisher’s exact test

### Primary outcome

**Fig. 3:**
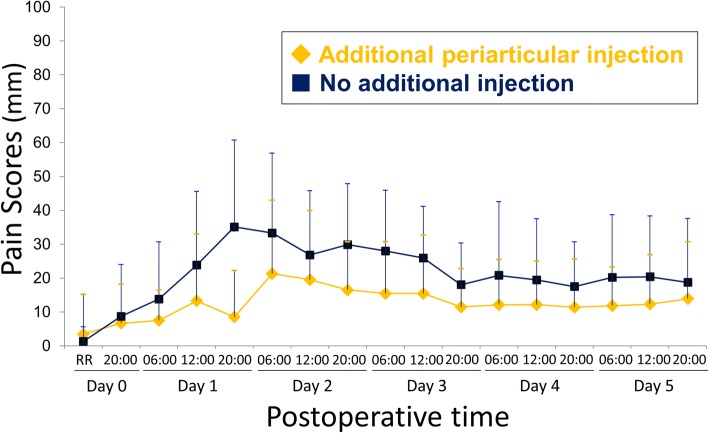
The mean and standard deviation visual analog scale scores for pain at rest after total knee arthroplasty**.** We injected the additional percutaneous periarticular multi-drug injection on postoperative day 1, at 08:30. The mean area under the curve was 1616 in the additional periarticular injection group compared with 2808 in the no additional injection group (95% confidence interval, − 2043 to − 340, *p* = 0.007). RR, recovery room

Figure 3 shows the mean value of postoperative pain VAS scores at rest. The additional periarticular injection group had significantly lower AUC of VAS score at rest than the no injection group (1616 ± 1191 compared with 2808 ± 1494, 95% confidence interval, − 2043 to − 340, *p* = 0.007).

The sphericity was rejected with Mauchly sphericity test (*p* < 0.001). Repeated measures analysis of variance with the Greenhouse-Geisser correction also showed that the effect of group (additional periarticular injection) was associated with the difference of VAS at rest (*p* = 0.04).

In the additional periarticular injection group, the mean pain VAS scores at rest were below the threshold value of the PASS of 33 mm in all assessment time points after TKA (Fig. 3). In the no additional injection group, the mean pain VAS score at rest exceeded the threshold value of the PASS of 33 mm at postoperative day 1, 20:00 and postoperative day 2, 06:00 and dropped below the threshold value of the PASS at the postoperative day 2, 12:00 (Fig. 3). Table 2 summarizes the number of patients whose VAS score was over the threshold values of PASS.

**Table 2:** The number of patients whose visual analog scale score was over the threshold values of patient acceptable symptomatic state of 33 mm

Measurement time	Additional periarticular injection (21 patients)	No additional injection (20 patients)
Postoperative day 0, recovery room	1	0
Postoperative day 0, 20: 00	1	2
Postoperative day 1, 06:00	0	2
Postoperative day 1, 12:00	3	5
Postoperative day 1, 20:00	1	9
Postoperative day 2, 06:00	7	9
Postoperative day 2, 12:00	5	5
Postoperative day 2, 20:00	2	9
Postoperative day 3, 06:00	2	5
Postoperative day 3, 12:00	2	5
Postoperative day 3, 20:00	1	2
Postoperative day 4, 06:00	2	4
Postoperative day 4, 12:00	1	4
Postoperative day 4, 20:00	1	4
Postoperative day 5, 06:00	2	6
Postoperative day 5, 12:00	2	6
Postoperative day 5, 20:00	2	4

The additional percutaneous periarticular multi-drug injection was routinely performed at postoperative day 1, 08:30

The difference between two groups reached 20 mm of the MCID only at 20:00, postoperative day 1. The mean VAS scores at postoperative day 1, 20:00 were 9 mm in the additional periarticular injection group and 35 mm in the no injection group, respectively.

### Secondary outcome

The additional periarticular injection group was associated with lower VAS during activity (*p* = 0.03) (Table 3). The consumption of diclofenac sodium suppository as rescue treatment was similar between the two groups (Table 4). No significant difference was seen in terms of the range of knee motion between two groups (Table 5).

**Table 3:** Visual analogue scale score for postoperative pain during activity

Duration after surgery	Additional periarticular injection (21 patients)	No additional injection (20 patients)
Postoperative day 1	24 ± 22	44 ± 27
Postoperative day 2	27 ± 23	36 ± 19
Postoperative day 3	19 ± 22	34 ± 19
Postoperative day 4	15 ± 15	26 ± 20
Postoperative day 5	18 ± 19	30 ± 20

Results are expressed as mean ± standard deviation

Repeated measures analysis of variance showed that the effect of group (additional periarticular injection) was associated with the difference of visual analogue scale during activity (*p* = 0.03)

**Table 4:** Mean number of suppositories used as rescue analgesia

Duration after surgery	Additional periarticular injection (21 patients)	No additional injection (20 patients)
On the night of surgery	0.05 ± 0.22	0.10 ± 0.31
Postoperative day 1	0.10 ± 0.30	0.30 ± 0.57
Postoperative day 2	0.19 ± 0.40	0.10 ± 0.31
Postoperative day 3	0.14 ± 0.36	0.15 ± 0.49
Postoperative day 4	0.05 ± 0.22	0
Postoperative day 5	0	0.10 ± 0.45

Repeated measures analysis of variance showed that the additional periarticular injection was not associated with the difference of the number of suppositories used as rescue analgesia VAS at rest (*p* = 0.55)

**Table 5:** Range of motion following total knee arthroplasty

Duration after surgery	Additional periarticular injection (21 patients)	No additional injection (20 patients)
Flexion angle*		
Postoperative day 1	71 ± 13	71 ± 13
Postoperative day 2	77 ± 10	80 ± 8
Postoperative day 3	87 ± 10	85 ± 9
Postoperative day 4	92 ± 9	90 ± 10
Postoperative day 5	94 ± 10	94 ± 9
Extension angle†		
Postoperative day 1	−8 ± 3	− 8 ± 5
Postoperative day 2	− 8 ± 3	− 9 ± 5
Postoperative day 3	−7 ± 3	− 8 ± 4
Postoperative day 4	−6 ± 4	−7 ± 4
Postoperative day 5	−6 ± 4	−6 ± 5

Results are expressed as means ± standard deviation

*Repeated measures analysis of variance showed that the additional periarticular injection was not associated with the difference of the flexion angle (*p* = 0.75)

† Repeated measures analysis of variance showed that the additional periarticular injection was not associated with the difference of the extension angle (*p* = 0.59)

No wound complication was recorded in any study patient. There were no patients that developed surgical site infection. In addition, no cardiac or central nervous system toxicity was observed in any study patient.

## Discussion

In this randomized trial, we evaluated the effectiveness of percutaneous periarticular multi-drug injection at the day following TKA as a component of multimodal pain management including intraoperative periarticular multi-drug injection. The percutaneous periarticular multi-drug injection was associated with significantly lower postoperative pain VAS score at rest. The mean pain VAS scores were under the threshold value of the PASS in the additional periarticular injection group in all assessment points. The difference of pain VAS score reached the MCID at 20:00, postoperative day 1. In addition, the pain VAS score during activity was less in the periarticular injection group.

The cumulative postoperative pain VAS score was significantly better in the additional periarticular injection group than no additional injection group. However, the difference of the mean pain VAS scores between groups reached the MCID of 20 mm only at 20:00, postoperative day 1. We believe that this short-duration difference may be meaningful for patients undergoing TKA since the time point of 20:00, postoperative day 1 generally corresponds to the peak of rebounding pain after intraoperative periarticular injection [4, 5].

Postoperative intraarticular local anesthetics through catheter has been applied to the multimodal pain management after TKA [2, 7, 8]. To our own knowledge, the first study of intraarticular multi-drug injection through catheter was reported by Ikeuchi et al.: they injected solution including local anesthetics, corticosteroid, and antibiotics every 12 h until 48 h after TKA [9]. The distinctive features of the technique used in this study were no placement of the infusion catheter and the injection into the muscle belly of vastus mediallis.

Our postoperative pain management regimen did not include any opioids. The United States is faced with the opioid epidemic [15, 16]. The excessive postoperative prescription of opioid medication has been one of the most crucial factors of the opioid epidemic [17]. Because a recent study revealed patients who underwent TKA tend to receive the highest amount of opioid medication among the major elective orthopedic surgeries [17], effective non-opioid pain management is important for patients undergoing TKA. We believe that our non-opioid pain management regimen may be an alternative regimen to the pain relief after TKA.

The most important limitation of our study is that the treatment team members and patients were not blinded. The non-blinding design is known to cause overestimation in randomized controlled trials [18]. Second, although this study showed a significant difference between groups in terms of primary outcome, the sample size was underpowered to provide conclusion whether percutaneous periarticular multi-drug injection would increase the postoperative complication. Third, the percutaneous periarticular multi-drug injection was routinely performed at postoperative day 1, 08:30 regardless of the time that TKA was performed. Thus, the periods of time between the completion of TKA and the injection were not standardized among study patients. Fourth, although the percutaneous periarticular multi-drug injection was painful procedure, we did not evaluate the pain provoked by this injection. Finally, we did not assess the accuracy of procedure of the percutaneous periarticular multi-drug injection.

The randomized, controlled design is the most important strength of our study. Another strength is the use of the remote method of randomization, in which the study institution is different from the site managing randomization schedule. We believe that the use of the AUC to analyses serial measurements of primary outcome may be the strength of this study since the AUC can avoid the serious problems associated with the use of comparisons at each time point when analyzing serial measurements on patients [11, 19, 20].

## Conclusion

The percutaneous periarticular multi-drug injection including methylprednisolone, ropivacaine, and epinephrine may have better postoperative pain relief than no additional injection after TKA. Further studies are needed to confirm the safety of the percutaneous periarticular multi-drug injection.

## Abbreviations

AUC: Area under the curve
MCID: Minimal clinically important difference
PASS: Patient acceptable symptomatic state
TKA: Total knee arthroplasty
VAS: Visual analog scale
